# Antiviral effects of mogroside V against porcine reproductive and respiratory syndrome virus *in vitro*

**DOI:** 10.3389/fmicb.2025.1611600

**Published:** 2025-07-02

**Authors:** Longhua Liang, Xiaohui Huang, Guoxi Qin, Xiaoyu Ma, Rijing He, Hongpeng Dai, Zhen Zhang, Xiaogan Yang, Xingwei Liang

**Affiliations:** ^1^College of Animal Science and Technology, Guangxi University, Nanning, China; ^2^Guangxi Key Laboratory of Animal Breeding & Disease Control and Prevention, Guangxi University, Nanning, China; ^3^Guangxi Agricultural Reclamation Yongxin Livestock Group Jinguang Livestock Co., Ltd, Nanning, China

**Keywords:** PRRSV, mogroside V, antiviral action, antioxidant, cytokines

## Abstract

Porcine reproductive and respiratory syndrome virus (PRRSV) infection has inflicted devastating impacts on the global swine industry, while current vaccines provide limited protection against this disease. Mogroside V (MV), a triterpenoid compound derived from Siraitia grosvenorii, exhibits diverse biological activities including antioxidant, anti-inflammatory, and anti-cancer properties, with the capacity to scavenge free radicals and mitigate oxidative stress. In this study, MV was administered to PRRSV-infected cells via three distinct treatment modalities. Our findings demonstrate that MV effectively blocks or suppresses infections caused by diverse PRRSV subtypes in porcine alveolar macrophages (PAMs) and Marc-145 cells. MV exhibited significant dose-dependent antiviral efficacy, with viral titers and mRNA expression inhibited by over 90% at a concentration of 400 μM. Comparative analysis further revealed substantial variations in antiviral efficacy among the different treatment protocols. Notably, PRRSV employs immune evasion mechanisms to suppress host innate immunity. MV not only directly inhibited PRRSV replication but also significantly upregulated the gene expression of immunomodulatory cytokines (IL-1, IL-2, IL-8, IL-18; *P* < 0.05), suggesting a dual mechanism of antiviral action. These findings underscore the antiviral bioactivity of MV and highlight its potential as a novel therapeutic candidate for PRRSV intervention.

## Introduction

Since its initial emergence in the late 1980s, porcine reproductive and respiratory syndrome virus (PRRSV) has caused substantial economic losses to the global swine industry, remaining a focal pathogen in porcine disease research to date (Wensvoort et al., 1991; Baron et al., 1992). PRRSV, classified under the family Arteriviridae, is an enveloped, single-stranded, positive-sense RNA virus with a genome of ~15 kb. It is categorized into two genotypes: PRRSV-1 (European type) and PRRSV-2 (North American type), which exhibit significant genomic and antigenic divergence (Hanada et al., 2005). In China, PRRSV-2 dominates the epidemiological landscape, primarily represented by four subtypes: Lineage 1 (epitomized by NADC30-like strains), Lineage 3 (QYYZ-like strains), Lineage 5 (VR2332-like strains), and Lineage 8 (highly pathogenic PRRSV, HP-PRRSV). Notably, Lineage 1 (NADC30-like strains) has emerged as the predominant circulating subtype in recent years (Zheng et al., 2025). PRRSV infection induces reproductive failure in pregnant sows (e.g., abortions, stillbirths, mummified fetuses) and respiratory diseases (e.g., pneumonia) across pigs of varying ages (Tong et al., 2007; Li et al., 2025). The virus primarily targets porcine alveolar macrophages (PAMs) for replication, suppresses host innate immune responses, and establishes persistent infections (Wang F. X. et al., 2021). The high genetic variability of PRRSV stems from the error-prone RNA-dependent RNA polymerase (RdRp), which lacks proofreading activity, facilitating frequent mutations and recombination during replication (Molitor et al., 1997; Zhang et al., 2022; Kong et al., 2023). Current PRRSV control strategies rely on vaccination, biosecurity protocols, and early diagnostics. However, existing vaccines confer limited protection and fail to fully prevent viral transmission or evolution (Sanhueza et al., 2023; Cui et al., 2024). Recent advances in molecular biology and immunology have introduced novel approaches, such as gene-edited PRRSV-resistant transgenic pigs and next-generation vaccine development (Xu et al., 2020; Chang H. et al., 2024). Despite these advancements, significant challenges persist, including incomplete immune protection and unresolved limitations in curbing pathogen spread.

Phytotherapeutic agents have garnered significant attention as potential alternative therapies due to their broad availability, minimal side effects, and low propensity for inducing drug resistance. Recent advances highlight their remarkable progress in combating porcine reproductive and respiratory syndrome virus (PRRSV). Numerous botanical extracts demonstrate potent anti-PRRSV activity. For instance, extracts from traditional medicinal herbs such as Astragalus membranaceus (Huangqi), Lonicera japonica (Jinyinhua), and Isatis indigotica (Banlangen) exert antiviral effects by suppressing viral replication (Chang W. et al., 2024). Studies have demonstrated that Astragalus polysaccharides significantly inhibit PRRSV replication and alleviate virus-induced inflammatory responses through modulation of host immune pathways (Chen et al., 2022). Bioactive phytochemicals, including flavonoids, polyphenols, and alkaloids, inhibit PRRSV replication via diverse mechanisms (Wang X. et al., 2021). Notably, quercetin impedes viral entry and replication processes (Guang et al., 2024), while epigallocatechin gallate (EGCG) from green tea blocks PRRSV infection by interfering with virus-host cell binding (Yu et al., 2022). Beyond direct antiviral activity, phytotherapeutic agents counter PRRSV by orchestrating host immunomodulation. Antioxidant compounds derived from plants mitigate PRRSV-induced oxidative stress, thereby attenuating tissue damage (Liu et al., 2024).

Siraitia grosvenorii, a traditional Chinese dual-purpose medicinal and edible plant, produces fruits rich in diverse bioactive compounds, with mogrosides being the most representative constituents (Liu et al., 2016). Mogroside V (MV), the predominant component among mogrosides, is a triterpenoid compound exhibiting multifaceted bioactivities, including antioxidant, anti-inflammatory, and anti-diabetic properties. Studies have demonstrated its potent antioxidant capacity to scavenge free radicals and alleviate oxidative stress (Pan et al., 2022; Cai Shi et al., 2023). Furthermore, MV exerts anti-inflammatory effects by suppressing the release of pro-inflammatory cytokines and mitigating inflammatory responses (Kim et al., 2023; Sung et al., 2023). In diabetes management, MV enhances insulin sensitivity and reduces blood glucose levels (Xiangyang et al., 2006; Qin et al., 2024). Notably, it demonstrates anti-metastatic activity against cancer cells (Chen et al., 2019; Wu et al., 2024). While significant advancements have been made in understanding MV's antioxidant, anti-inflammatory, and anticancer properties, no studies have yet reported its antiviral activity.

Porcine reproductive and respiratory syndrome virus (PRRSV) poses severe threats to swine health and inflicts substantial economic losses, particularly in high-density farming regions. Consequently, identifying effective antiviral agents against PRRSV is imperative. This study aimed to investigate the antiviral efficacy of mogroside V (MV) against PRRSV. Through *in vitro* antiviral assays, we evaluated the inhibitory effects of MV on PRRSV infection. The results demonstrated that MV significantly suppressed PRRSV replication in cultured cells, as evidenced by reduced viral titers and inhibited viral mRNA expression. These findings suggest that MV holds promise as a therapeutic candidate for PRRSV, with substantial potential for development into novel veterinary therapeutics.

## Materials and methods

### Viruses and cells

The permissive cell line Marc-145 was maintained in the Veterinary Medicine Laboratory of Guangxi University. Porcine alveolar macrophages (PAMs) were aseptically isolated from PRRSV-negative healthy piglets. The cells were cultured at 37°C in a 5% CO_2_ incubator and grown in Dulbecco's modified Eagle's medium (DMEM, Wisent) supplemented with 10% fetal bovine serum (FBS, Gibco). This study utilized two PRRSV strains preserved in the aforementioned laboratory: the currently prevalent NADC30 strain and a high-pathogenicity PRRSV (HP-PRRSV) strain engineered to express the green fluorescent protein (GFP) gene (designated HP-PRRSV-GFP). Both NADC30 and HP-PRRSV-GFP strains were propagated in PAMs and Marc-145 cells. Viral amplification and titration were performed in Marc-145 cells, yielding titers of 10^5.1^ TCID_50_/mL for the NADC30 strain and 10^4.8^ TCID_50_/mL for the HP-PRRSV strain. The TCID_50_ represents the viral dilution required to infect 50% of inoculated cell cultures. This quantitative virological analysis measures infectious virus titers in tissue culture systems using the Reed-Muench method.

### Mogroside V

Mogroside V (MV) was obtained from Chengdu Mansite Biotechnology Co., Ltd. (China) with a certified purity of ≥98%. The lyophilized MV powder was stored at −20°C under desiccation and reconstituted in sterile double-distilled water (ddH_2_O) immediately prior to experimentation. To preserve stability, working solutions were aliquoted to minimize repeated freeze-thaw cycles.

### Cytotoxicity assay

The cytotoxicity of MV was assessed using the Cell Counting Kit-8 (CCK-8) assay to determine cell viability. Porcine alveolar macrophages (PAMs) and Marc-145 cells were seeded in 96-well plates and treated with varying concentrations of MV (0, 100, 200, 400 or 800 μM) for 24, 48, 72, and 96 h. Following the manufacturer's protocol, CCK-8 reagent was added to each well, and cells were incubated for 2 h at 37°C under 5% CO_2_. Absorbance was measured at 450 nm using a microplate reader. Cell viability (%) was calculated using the formula:Cell viability (%) = [OD (sample) – OD (blank)/OD (control) – OD (blank)] × 100%, where OD (blank) represents the background absorbance of cell-free medium, and OD (control) denotes untreated cells.

### Antiviral assays

To investigate the stage-specific antiviral effects of mogroside V (MV) and evaluate its ability to inhibit PRRSV replication in Marc-145 and PAM cells, three distinct treatment groups were established for *in vitro* evaluation: pre-treatment group, post-treatment group, and co-treatment group. Viral replication was assessed through multiple approaches: quantification of viral mRNA copies by RT-qPCR, determination of viral titers using the TCID_50_ method, and analysis of PRRSV N protein expression via immunofluorescence assay (IFA). The experimental design was as follows:

Pre-treatment group: to investigate the preventive effect of MV prior to PRRSV infection, Marc-145 or PAM cells were pretreated with MV before viral challenge. Cells in good growth condition were seeded in 6-well plates and cultured until monolayer formation. After removing the old medium, cells were washed twice with PBS, followed by incubation with varying concentrations of MV for 24 h. The drug was then removed, and cells were washed twice with PBS before infection with PRRSV (100 TCID_50_) for 48 h. Subsequently, viral mRNA copies, viral titers, and PRRSV N protein fluorescence expression were measured to calculate viral inhibition rates.

Post-treatment group: In order to investigate the effect of MV on PRRSV infection, MV was added after Marc-145 or PAM cells were infected with PRRSV. The cells with good growth status were inoculated in the 6-well cell culture plate. After the cells grew to monolayer, the old medium was discarded and the cells were cleaned twice with PBS. PRRSV (100 TCID_50_) was inoculated and incubated for 1 h, during which the virus could be shaken repeatedly to fully interact with cells. The virus venom was removed, washed twice with PBS, and incubated with different concentrations of MV for 48 h. After that, the viral mRNA copies and viral titer were detected, the viral inhibition rate was calculated, and the fluorescence expression of PRRSV N protein was observed.

Co-treatment group: In order to study the direct effect of MV on PRRSV, PRRSV and MV were simultaneously inoculated in Marc-145 or PAM cells for treatment. The cells with good growth status were inoculated in the 6-well cell culture plate. After the cells grew to monolayer, the old medium was discarded and the cells were cleaned twice with PBS. MV at different concentrations was mixed with PRRSV (100 TCID_50_) and inoculated into Marc-145 and PAM cells for 48 h. After that, the viral mRNA copies and viral titer were detected, viral inhibition rate was calculated, and the fluorescence expression of PRRSV N protein was observed.

### Dose–response and IC_50_ value estimation

The half-maximal inhibitory concentration (IC_50_) value for MV-mediated cytotoxicity was calculated through nonlinear regression fitting of dose-response curves.

### Real-time quantitative PCR

Real-time quantitative PCR (RT-qPCR) was performed to determine PRRSV gene expression levels. Viral RNA was extracted from cell culture supernatants using a commercial viral DNA/RNA extraction kit according to the manufacturer's protocol, with subsequent storage at −80°C until further analysis. RT-qPCR amplification were conducted using TransScript Green One-Step qRT-PCR SuperMix kit. The relative expression levels of cellular cytokines were analyzed by RT-qPCR with primer sequences listed in Table 1.

**Table 1:** Primers probes used in this study.

**Genes**	**Primers**	**Sequences(5^′^-3^′^)**	**Nucleotide (nt)**
IL−1	F	TTCAAATCAGCCGCCCATCCAAAG	24
IL−1	R	ACAGGTAAGTAGACACCAGAGTCAAGAC	28
TNF-a	F	CCCTGATTTCTAAGTGTTGC	20
TNF-a	R	CTGCCCGACTATCTGGAC	18
IL−8	F	AGTTTTCCTGCTTTCTGCAGCT	22
IL−8	R	TGGCATCGAAGTTCTGCACT	20
β-actin	F	CCATCGTCCACCGCAAAT	18
β-actin	R	CCAAATAAAGCATGCCAATC	20
IL−6	F	CTGGCAGAAAACAACCTGAACC	22
IL−6	R	TGATTCTCATCAAGCAGGTCTCC	23
IL-β	F	CCCAAAAGTTACCCGAAGAGG	21
IL-β	R	TCTGCTTGAGAGGTGCTGATG	21
IL−2	F	GAGCCATTGCTGCTGGATTT	20
IL−2	R	GTAGCCTGCTTGGGCATGTAA	21
IFN-a	F	CTGGCACAAATGAGGAGAAT	20
IFN-a	R	TGCTGAAGAGCTGGAAGGT	19
IFN-β	F	TGCAACCACCACAATTCC	18
IFN-β	R	CTGAGAATGCCGAAGATCTG	20
IL-18	F	GCAGTAACCATCTCTGTGCAGTGTA	25
IL-18	R	TCATCAATATTATCAGGAGGACTCATTT	28
IP– 10	F	CAGAACTGTTCGCTGTACC	19
IP– 10	R	CATGTGGGCAAGATTGAC	18

### Immunofluorescence assay

The supernatant from 6-well culture plates was collected into 1.5 mL microcentrifuge tubes and stored at −80°C for subsequent analysis. Cells were washed twice with PBS and fixed with ice-cold methanol for 15 min at room temperature. Non-specific binding sites were blocked with 1% BSA containing 0.05% Tween 20 for 30 min at ambient temperature. Cells were then incubated with anti-PRRSV N protein monoclonal antibody either for 2 h at 37°C or overnight at 4°C. Following primary antibody incubation, cells were treated with fluorophore-conjugated secondary antibody (goat anti-mouse IgG, Alexa Fluor 488) for 1 h at 37°C. Nuclei were counterstained with DAPI for 10 min at room temperature protected from light. Fluorescence microscopy was performed to examine the subcellular distribution and expression levels of PRRSV N protein. Stained cell monolayers were visualized under 4 × , 10 × , and 20 × objective lenses using an epifluorescence microscope. Representative digital images were acquired using camera integrated with the microscopy system.

### Statistical analysis

All experimental data are presented as mean ± standard deviation (SD). Raw data were collated using Microsoft Excel and subjected to statistical analysis using SPSS 29.0 software. Intergroup differences were evaluated by one-way analysis of variance (ANOVA) followed by appropriate *post-hoc* tests. The threshold for statistical significance was set at *P* < 0.05. Significant differences among groups are denoted by lowercase superscript letters (a, b, c), where: Groups sharing the same letter indicate no statistically significant difference (*P* > 0.05), Groups with different letters demonstrate significant differences (*P* < 0.05). Graphical representations were generated using GraphPad Prism 8.0 software with error bars representing SD. All experiments were performed with a minimum of three biological replicates.

## Results

### Cytotoxicity of MV against PAM and Marc-145 cells

**Figure 1:**
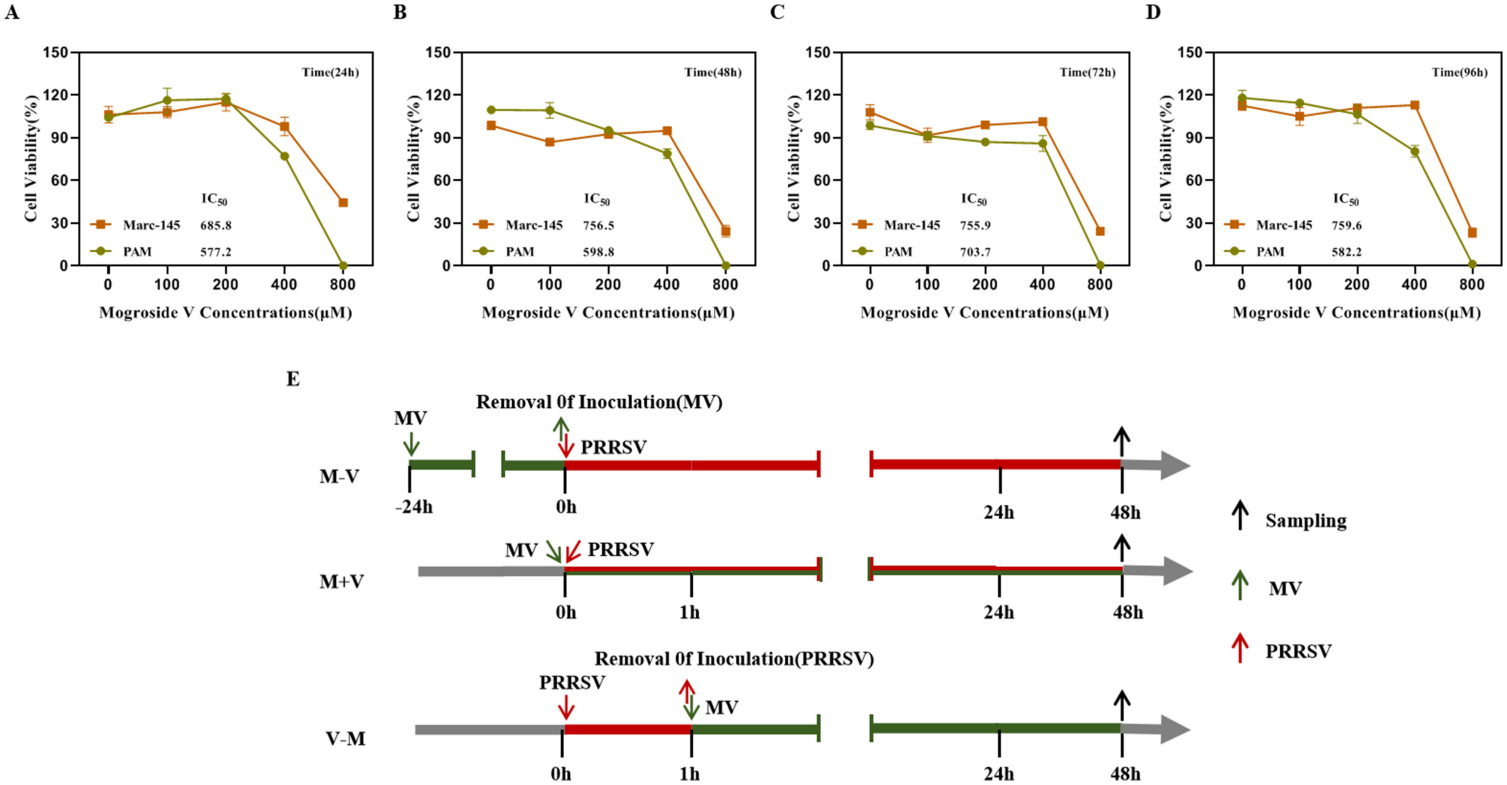
Cytotoxicity of MV against PAM and Marc-145 cells. **(A–D)** The cytotoxic activities of Mogroside V (MV) at four different treatment times (24h, 48h, 72h and 96h) against Marc-145 and PAM cells were assessed using a CCK-8 assay. IC50, the con-centration for 50 % inhibition of the maximal effect. **(E)** Experimental schematic and timeline of the virus inhibition experiment. The maximum concentration of MV added in subsequent experiments was 400 μM. (M-V) Drug pretreatment: MV was added to cells at 37°C for 24 h. The cells were then washed twice with PBS and replenished with virus-containing maintenance medium, followed by incubation at 37°C for 48 h. (V-M) Drug post-treatment (Blocking): After viral infection for 1 hour, the virus was removed, and the cells were washed twice with PBS. The cells were then supplemented with MV-containing maintenance medium and incubated for 48 h. (M+V) Co-treatment: MV and the virus were simultaneously added to the cells and incubated for 48 h.

The cytotoxicity of MV was evaluated using the CCK-8 assay. Marc-145 and PAM cells were treated with varying concentrations of MV (0, 100, 200, 400, and 800 μM), alongside a negative control group (untreated cells) and a blank control group (cell-free medium), followed by incubation for 24, 48, 72, and 96 h. The half-maximal inhibitory concentration (IC_50_) of MV in Marc-145 and PAM cells was determined. At 24 h, the IC_50_ values were 685.8 μM and 577.2 μM (Figure 1A), respectively; at 48 h, they were 756.5 μM and 598.8 μM (Figure 1B); at 72 h, they were 755.9 μM and 703.7μM (Figure 1C); and at 96 h, they were 759.6 μM and 582.2 μM (Figure 1D). The IC_50_ values were all ≥ 577.2 μM. The results demonstrated that at an MV concentration of 400 μM, the viability of both PAM and Marc-145 cells exceeded 75%, indicating no significant cytotoxicity. Therefore, 400 μM was established as the maximum safe drug concentration for subsequent antiviral studies.

### Antiviral effects of MV against two PRRSV strains

**Figure 2:**
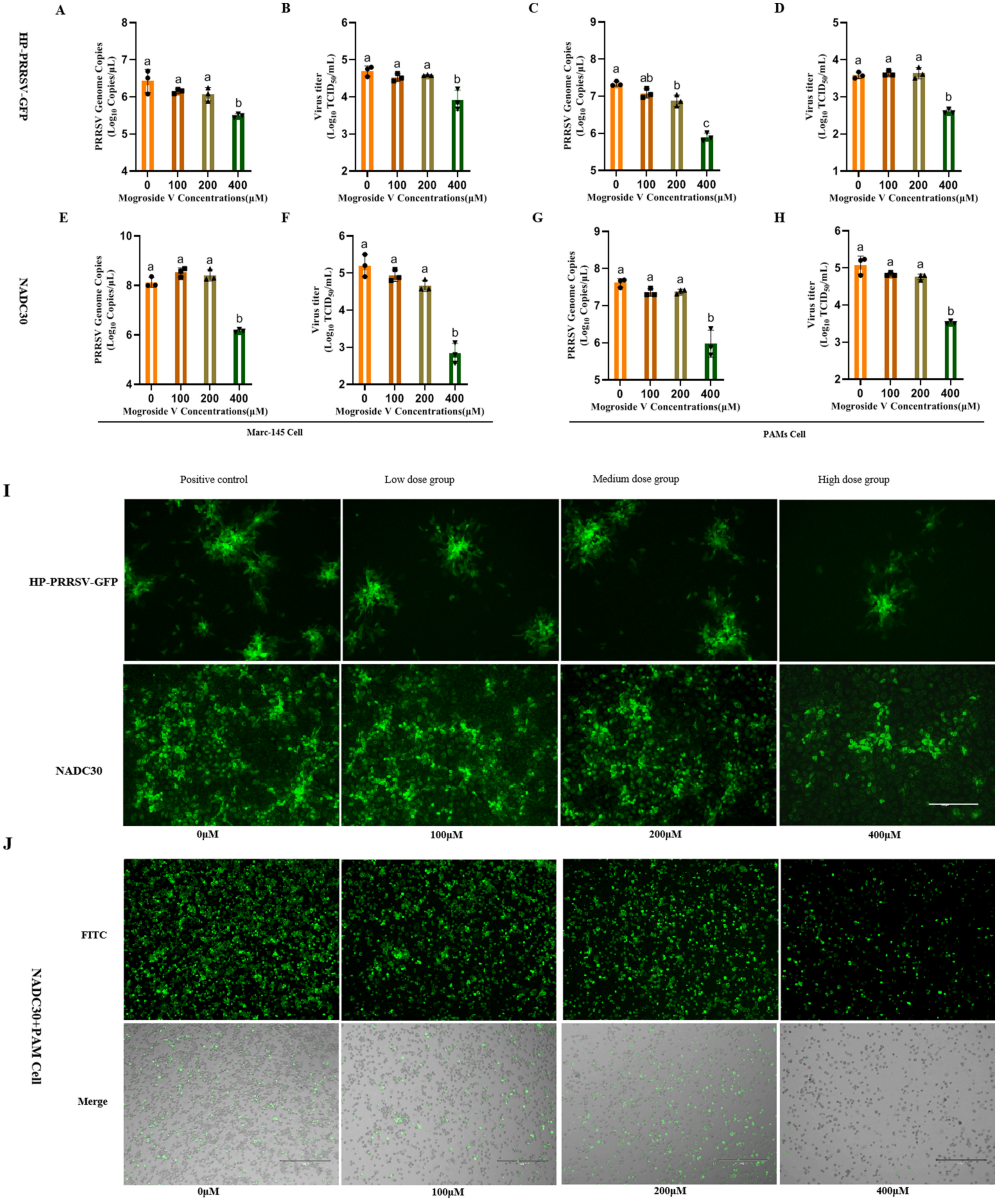
Antiviral Effects of MV Against Two PRRSV Strains. **(A–H)** MV (0, 100, 200 and 400 μM) was added to Marc-145 and PAM cell monolays and incubated for 24 h, followed by PRRSV virus (HP-PRRSV-GFP and NADC30 strains,100TCID50), 37°C after incubation for 48 h, the cell supernatant was collected. Viral mRNA copies was detected by RT-qPCR and viral titer was detected by TCID50. The data are expressed as the mean ± standard deviation (SD) for a sample size of 3 (*n* = 3). **(I)** Results of IFA in Marc-145 cells infected with the two PRRSV strains, with three replicates treatment. **(J)** IFA results of the NADC30 strain in PAMs. All images were taken at 10× objective magnification, scale bar is set at 300 μm. Values marked with different letters (a, b, c) indicate statistically significant differences (*P* < 0.05), the same letters had no statistical significance (*P* > 0.05).

In this study, Marc-145 and PAM cells were treated with escalating concentrations of MV (0, 100, 200, and 400 μM) prior to infection with two PRRSV strains (NADC30 and HP-PRRSV-GFP) at a standardized inoculum of 100 TCID_50_. Viral replication was assessed 48 h post-infection (hpi) through integrated virological and molecular analyses. MV exhibited suppression of viral protein expression and replication across both PRRSV subtypes, as validated by TCID_50_ determine, RT-qPCR, and IFA (Figure 2).

Notably, 400 μM MV significantly reduced viral titers and PRRSV mRNA levels in both cell types compared to untreated controls (*P* < 0.01; Figures 2A–H). In Marc-145 cells, HP-PRRSV-GFP titers decreased from 10^4.7^ to 10^3.9^ TCID_50_/mL (Figure 2B), while NADC30 titers declined from 10^5.2^ to 10^2.8^ TCID_50_/mL (Figure 2F). Similar trends were observed in PAMs: HP-PRRSV-GFP titers dropped from 10^3.6^ to 10^2.6^ TCID_50_/mL (Figure 2D), and NADC30 titers decreased from 10^5.1^ to 10^3.5^ TCID_50_/mL (Figure 2H). At 200 μM, MV significantly suppressed HP-PRRSV-GFP mRNA copies in PAMs (*P* < 0.05; Figure 2C), though no significant reduction in viral titers was observed (P >0.05; Figure 2D). IFA corroborated these findings, in Marc-145 cells, high-dose MV (400 μM) markedly diminished viral fluorescence intensity (*P* < 0.01; Figure 2I). Similarly, PAMs treated with 400 μM MV exhibited significantly reduced PRRSV N protein expression (*P* < 0.01; Figure 2J), whereas lower concentrations (100 and 200 μM) showed no statistically significant effects. Collectively, these data establish MV as a potent inhibitor of PRRSV replication in both Marc-145 and PAM cell models, with efficacy related to the concentration of MV.

### Differential antiviral efficacy of MV administration modalities against PRRSV

To gain a deeper understanding of the mechanism underlying MV-mediated PRRSV inhibition, we systematically compared two therapeutic strategies: post-treatment (V–M) and co-treatment (M+V), relative to pre-treatment (M–V). Marc-145 cells and PAMs were exposed to 100 TCID_50_/mL of NADC30 or HP-PRRSV-GFP strains with concurrent or sequential MV administration (0, 100, 200, 400 μM) (Figure 1E). Following 48 h of drug treatment, viral supernatants were harvested, and viral titers as well as viral mRNA copies were quantified using TCID_50_ assay and RT-qPCR, respectively. Concurrently, IFA was performed on the cells for viral validation.

**Figure 3:**
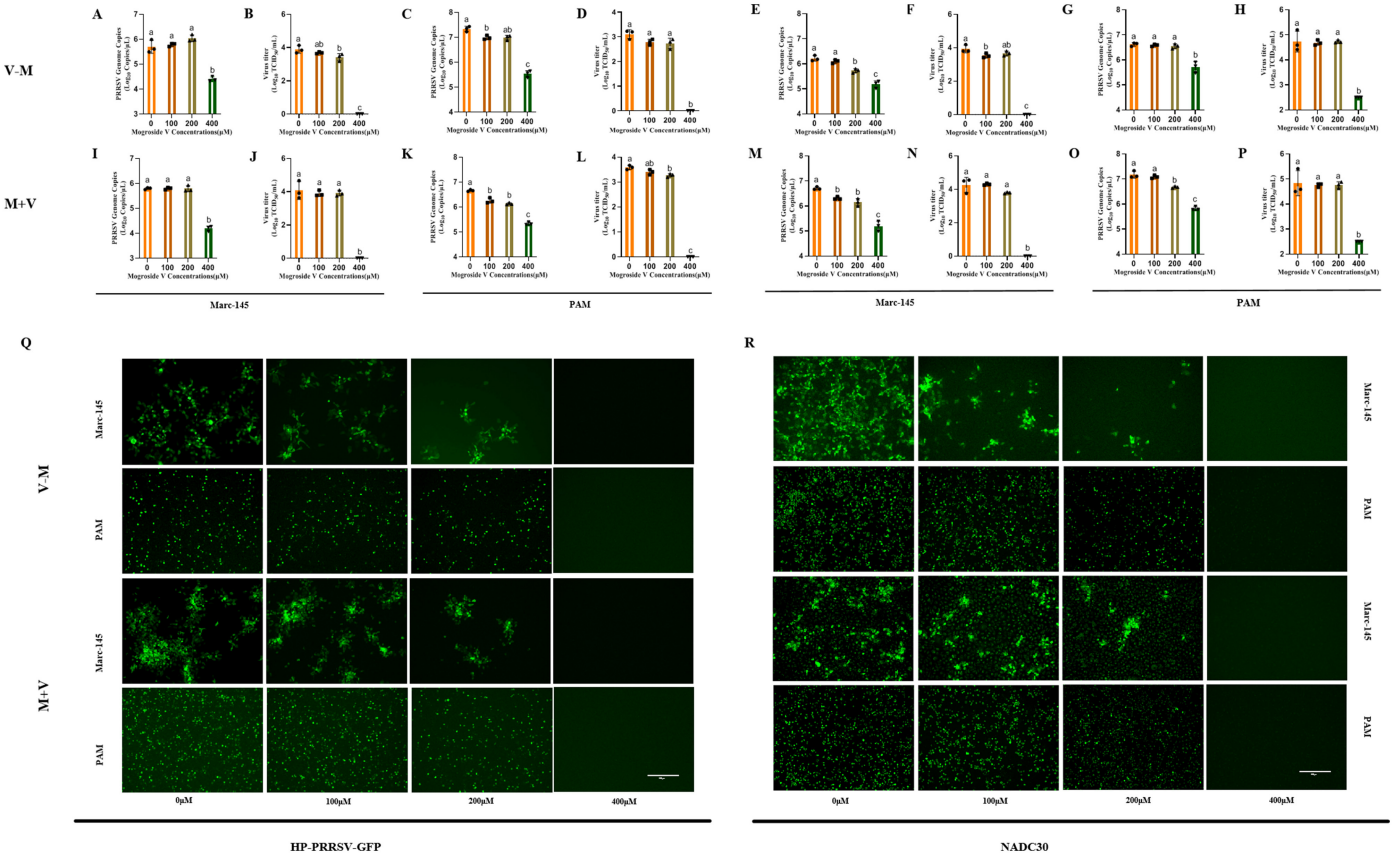
Differential Antiviral Efficacy of MV Administration Modalities Against PRRSV. **(A–H)** PRRSV (100 TCID50) was added to Marc-145 and PAM cell monolayer and incubated for 1h. MV (0, 100, 200 and 400 μM) 37°C was added and incubated for 48 h. The cell supernatant was collected to detect viral mRNA copies by RT-qPCR and viral titer by TCID50. **(I–P)** PRRSV (100 TCID50) and MV (0, 100, 200 and 400 μM) were added to Marc-145 and PAM cell monolays at the same time. After incubation at 37°C for 48 h, the cell supernatant was collected and the viral mRNA copies was detected by RT-qPCR and viral titer was measured by TCID50. The data are expressed as the mean ± standard deviation (SD) for a sample size of 3 (*n* = 3). **(Q)** IFA validation of the inhibitory effect of MV on the HP-PRRSV-GFP strain in Marc-145 and PAM cells. **(R)** IFA validation of the inhibitory effect of MV on the NADC30 strain in Marc-145 and PAM cells. All images were taken at 10x objective magnification, scale bar is set at 300 μm. Values marked with different letters (a, b, c) indicate statistically significant differences (*P* < 0.05), the same letters had no statistical significance (*P* > 0.05).

The results demonstrated that treatment with MV at varying concentrations initiated 1 h post-PRRSV infection led to a concentration-dependent suppression of viral replication. Specifically, at 400μM MV, viral mRNA copies were significantly reduced (P < 0.01), and viral titers decreased to undetectable levels, indicating complete inhibition of infectious viral replication under these experimental conditions (Figures 3A–H). Similarly, when MV was co-administered with PRRSV across a range of concentrations, the 400 μM MV treatment markedly suppressed viral replication in cells (*P* < 0.01) (Figures 3I–P). Specifically, in HP-PRRSV-GFP-infected Marc-145 and PAM cells, as well as NADC30-infected Marc-145 cells, viral titers dropped below the detection threshold (Figures 3J, L, N). In NADC30-infected PAM cells, viral titers approached the lower detection limit (CPE observed in only two replicate wells in TCID_50_ assays), with minimal residual infectivity (Figure 3P). Notably, immunofluorescence assay (IFA) revealed no detectable PRRSV N protein expression in either Marc-145 or PAM cells treated with 400 μM MV under both post-infection and co-treatment regimens (Figures 3Q, R). These findings indicate that both MV-mediated blockade (post-infection treatment) and co-treatment strategies exhibited comparable efficacy in suppressing PRRSV replication, effectively inactivating the virus at sufficient concentrations. Furthermore, both strategies demonstrated superior inhibitory efficacy compared to prophylactic treatment (M–V). Collectively, the study validates that MV inhibits PRRSV replication in a treatment modality-dependent manner.

### Dose-dependent inhibitory effect of MV on PRRSV replication

In this study, three concentrations of MV (100, 200, and 400 μM) were applied to Marc-145 and PAM cells under three treatment regimens (M–V, V–M, M+V). Viral inhibition rates for both viral titers (TCID_50_) and viral mRNA copies (RT-qPCR) were calculated using the formula: Inhibition rate = [1 – (Viral load in MV-treated group/Viral load in control group)] × 100%.

**Figure 4:**
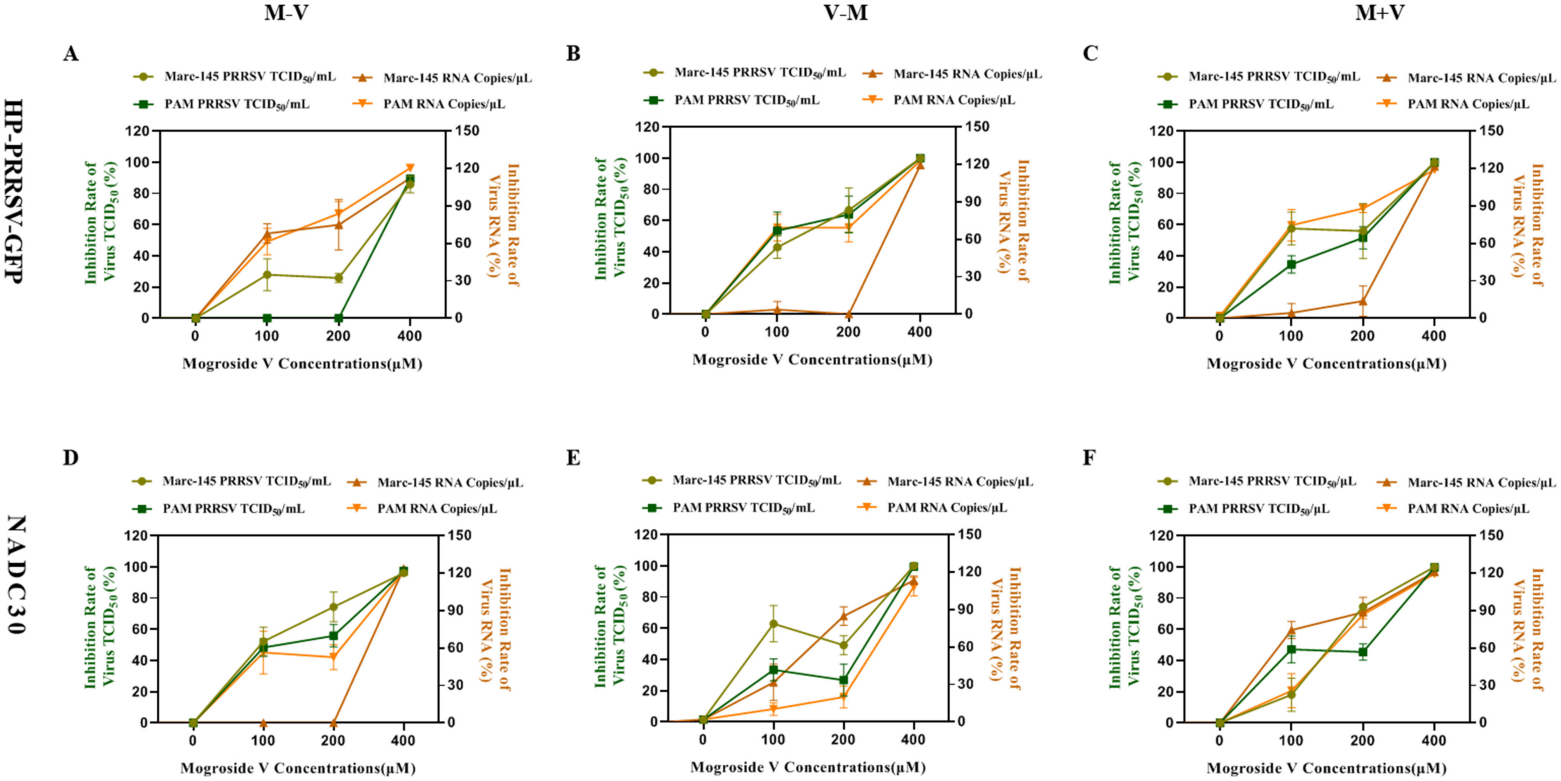
Dose-dependent inhibitory effect of MV on PRRSV replication **(A–C)** Viral inhibition rate of MV against the HP-PRRSV-GFP strain in Marc-145 and PAM cells. **(D–F)** Viral inhibition rate of MV against the NADC30 strain in Marc-145 and PAM cells. The left Y-axis represents the inhibition rate of PRRSV viral titer (TCID50), while the right Y-axis indicates the inhibition rate of PRRSV viral mRNA copies. The error bar represents the standard deviation of the three repeated measurements in the representative experiment.

For HP-PRRSV-GFP-infected Marc-145 and PAM cells, treatment with MV at concentrations ≤ 200 μM resulted in average mRNA inhibition rates below 42% and viral titer inhibition rates below 43%. In contrast, the 400 μM MV treatment achieved average mRNA and viral titer inhibition rates exceeding 95%. Notably, the high-dose group (400 μM) exhibited a 53-percentage-point increase in mRNA inhibition and a 52-percentage-point increase in viral titer inhibition compared to the medium-dose group (200 μM) (Figures 4A–C). Similarly, in NADC30-infected Marc-145 and PAM cells, MV concentrations ≤ 200 μM yielded average mRNA inhibition rates below 45% and viral titer inhibition rates below 55%. However, the 400 μM MV treatment elevated these values to >94% and >98%, respectively. The high-dose group demonstrated a 49-percentage-point enhancement in mRNA inhibition and a 43-percentage-point improvement in viral titer inhibition relative to the medium-dose group (Figures 4D–F).

Critically, across both viral strains (HP-PRRSV-GFP and NADC30), treatment with 400 μM MV consistently suppressed viral mRNA and viral titers in Marc-145 and PAM cells, with average inhibition rates surpassing 94%. These findings unequivocally demonstrate that MV inhibits PRRSV replication in a dose-dependent manner, with maximal efficacy achieved at the highest concentration tested.

### Modulatory effects of MV on gene expression of multiple immunoregulatory factors

**Figure 5:**
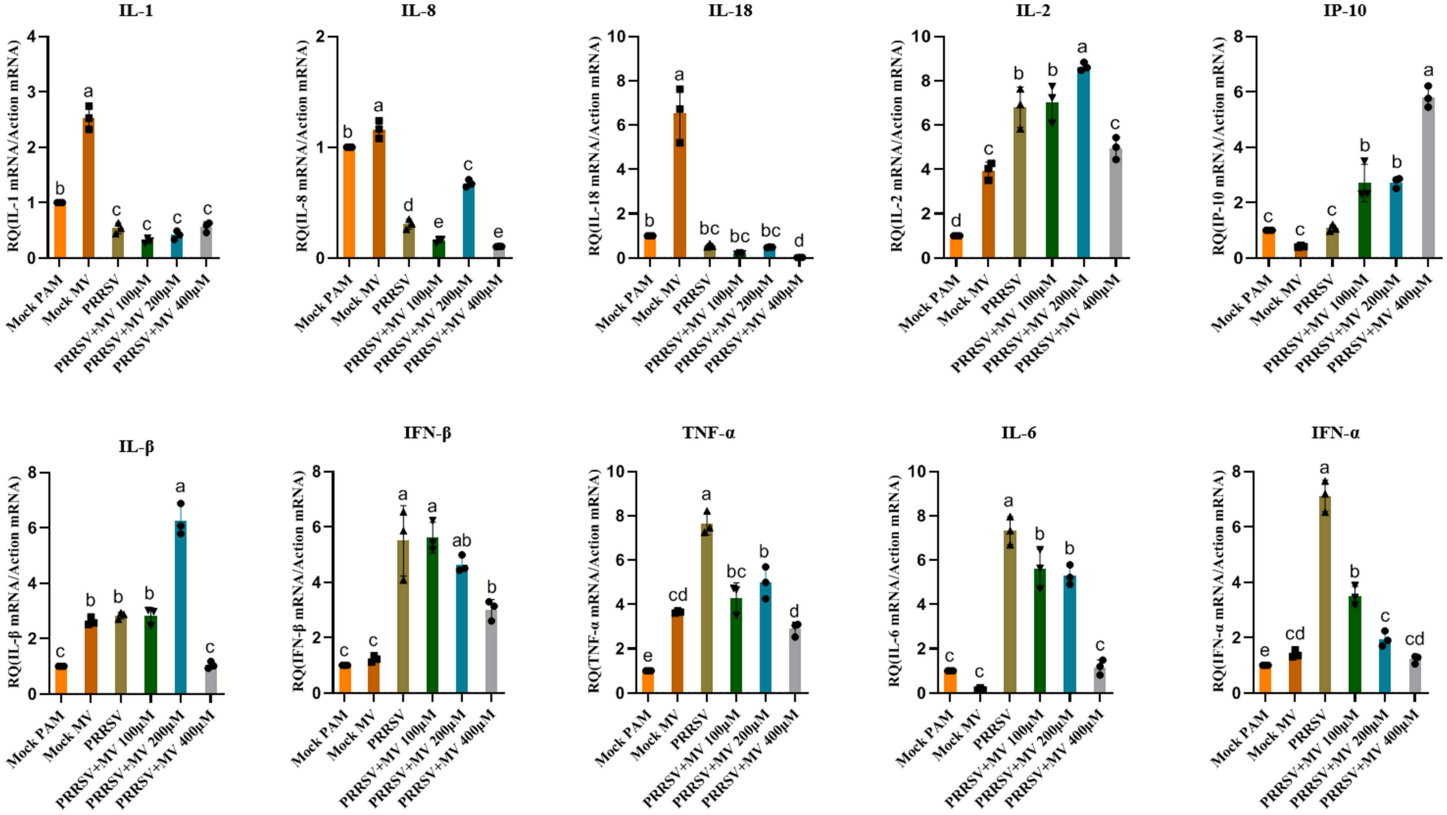
Modulatory effects of MV on gene expression of multiple immunoregulatory factors. The relative mRNA expression of PRRSV-induced proinflammatory cytokines in PAMs was evaluated by RT-qPCR. Statistical significance was determined by one-way ANOVA, with bar graph data presented as mean ± standard error (SE) from at least three independent experiments. Values marked with different letters (a, b, c) indicate statistically significant differences (*P* < 0.05), the same letters had no statistical significance (*P* > 0.05).

Our findings demonstrate that MV significantly upregulates the gene expression of key immunomodulatory factors, including IL-1, IL-2, IL-8, IL-18, and IL-1β (Figure 5). Beyond its direct antiviral activity against PRRSV replication, MV enhances host defense mechanisms by activating immune response pathways and upregulating critical immunoregulatory factors. This dual mode of action—combining viral suppression and immune potentiation—likely facilitates early cellular recognition of viral invasion, thereby augmenting the cell's intrinsic antiviral capacity (Ruan et al., 2024).

## Discussion

This study aimed to evaluate the antiviral efficacy of MV against PRRSV. MV exhibited potent inhibition against both the prevalent NADC30 strain and the GFP-tagged HP-PRRSV-GFP strain. At 400 μM, MV completely abolished viral infectivity, achieving up to 100% inhibition of viral titers, without inducing significant cytotoxicity (cell viability >75%). Notably, a viral inhibition rate ≥50% (indicating a halving of viral replication) is generally considered biologically meaningful, while ≥70% inhibition reflects strong antiviral potency—comparable to effective COVID-19 therapies that reduce viral loads by >1 log_10_ (Mazzotta et al., 2023). A 1-log_10_ reduction in viral titer (~10-fold) is clinically relevant, and robust antivirals typically suppress titers to undetectable levels (Hayden et al., 1999).

Critically, post-infection treatment (MV applied after viral adsorption) and co-treatment (MV administered concurrently with PRRSV) demonstrated superior inhibitory effects compared to prophylactic pretreatment (MV added pre-infection). This suggests that MV's anti-PRRSV mechanism primarily involves direct virion targeting or blockade of early viral entry steps, rather than host cell preactivation or sustained immunomodulation. In post-infection and co-treatment regimens, viral titers (TCID_50_) were reduced to undetectable levels, implying that MV may disrupt PRRSV structural integrity, thereby neutralizing infectivity. This aligns with known mechanisms of surfactants and phytochemicals that inactivate enveloped viruses like PRRSV via lipid membrane destabilization (Cheng et al., 2013). Under co-treatment conditions, MV likely alters host membrane properties, interfering with the microenvironment required for viral entry and subsequent replication (Jarvis et al., 2022). In contrast, prophylactic pretreatment may permit MV metabolism or efflux before viral challenge, allowing recovery of potential receptor targets prior to infection (Lagrange et al., 2013). These findings not only establish MV as a potent PRRSV inhibitor but also delineate treatment modality-dependent efficacy, highlighting its direct antiviral action over indirect host-mediated strategies.

The concentration of MV employed in this study proved critical for its antiviral efficacy. If the concentration used fell below the minimum inhibitory concentration (MIC) required for effective PRRSV suppression, MV's antiviral activity remained undetectable. A dose-dependent response was observed in PRRSV-infected Marc-145 and PAM cells: at 200 μM MV, inhibition rates for viral titers and mRNA reached 43 and 59%, respectively, whereas 400 μM MV achieved 97 and 94% inhibition (mean values across all treatment groups). The high-concentration group exhibited 48% and 51% greater suppression of viral titers and mRNA, respectively, compared to the low-concentration group. This aligns with the characteristic dose-response patterns of phytochemicals, which often exert multi-target antiviral effects. For instance, quercetin demonstrates dose-dependent suppression of Zika virus replication in A549 and Vero cells, reducing both viral titers and mRNA expression (Saivish et al., 2023). Such concentration-effect relationships may involve not only direct inhibition of viral replication but also modulation of host immune factors (Liu et al., 2021).

PRRSV infection is known to suppress innate immune responses in antigen-presenting cells (APCs), leading to delayed adaptive immunity and persistent viral replication (Charerntantanakul and Fabros, 2018). Significantly, MV treatment stimulated immune activation in porcine alveolar macrophages (PAMs), markedly upregulating the expression of immunomodulatory genes, including IL-1, IL-2, IL-8, IL-18, and IL-1β. These cytokines play pivotal roles in antiviral immunity by enhancing viral recognition, replication blockade, and pathogen clearance (Aarreberg et al., 2019). 25-Hydroxycholesterol significantly suppresses PRRSV replication while concurrently enhancing the production of IL-1β and IL-8 in porcine primary alveolar macrophages and lung tissues (Song et al., 2019). IL-1β plays a pivotal role in innate immunity and is critically important for host defense against viral infections (Wang et al., 2018). Stimulation by IL-1β subsequently enhances ADAM17 expression at both the transcriptional and protein levels. ADAM17, a crucial membrane-associated metalloprotease, primarily mediates inflammation and other pathological processes (Lanaya et al., 2014; Xu et al., 2017). Importantly, ADAM17 inhibits PRRSV entry by downregulating the expression of membrane CD163, a key viral receptor essential for mediating PRRSV entry and uncoating (Guo et al., 2014). Studies demonstrate that inflammasome activation in the airway epithelium, along with the production of IL-1β and IL-18, is essential for optimal antibody and T cell responses against Influenza A Virus (IAV). Furthermore, triterpenoid compounds can activate caspase-1 and induce IL-1β maturation via an IFI16-dependent pathway, thereby inhibiting PRRSV replication. Moreover, elevated levels of IL-1β production reduce the pathogenicity of PRRSV in infected pigs, enhancing viral clearance rates and improving survival rates among infected hosts (Zhang et al., 2022). Collectively, our findings position MV as a potent dual-function agent, combining direct antiviral activity with immunostimulatory properties that reinforce innate defenses against PRRSV.

PRRSV infection induces tissue damage and reproductive failure in sows and respiratory disease across all swine age groups by triggering reactive oxygen species (ROS) overproduction, suppressing antioxidant enzyme activity, and disrupting redox homeostasis, thereby provoking oxidative stress (Wang et al., 2025). Phytochemicals can mitigate PRRSV-induced oxidative stress through multifaceted mechanisms. For instance, xanthohumol upregulates antioxidant-related gene expression in Marc-145 cells, inhibits PRRSV replication in both Marc-145 and porcine alveolar macrophages (PAMs) via activation of the Nrf2-HMOX1 pathway, and attenuates virus-triggered oxidative damage (Liu et al., 2019). Notably, MV has demonstrated significant antioxidant capacity in prior studies, functioning through direct free radical scavenging, activation of the Nrf2-mediated antioxidant pathway, and upregulation of endogenous antioxidant enzymes (Mo et al., 2021). Furthermore, MV protects against corticosterone-induced oxidative injury and apoptosis in PC12 cells, underscoring its cytoprotective potential (Liu et al., 2023).

While MV exhibits considerable promise as an anti-PRRSV agent, its therapeutic development faces critical challenges. First, mechanistic elucidation remains incomplete: the precise molecular targets underlying its direct antiviral activity and immunomodulatory effects are yet to be defined. Second, current evidence is limited to *in vitro* models, with a paucity of *in vivo* validation in swine. Research has demonstrated that mice administered Luo Han fruit concentrate at 100,000 ppm for 28 days exhibited no significant adverse reactions, indicating a lack of overt toxicity for both the whole fruit and its extract in animal models (Marone et al., 2008). Furthermore, MV selectively upregulates antiviral cytokines (e.g., IL-2, IL-8). Critically, the experimental results showed no evidence of a pro-inflammatory cytokine storm, such as excessive TNF-α secretion, thereby preventing the onset of immunopathological damage. This profile suggests MV is particularly safe for PRRSV-susceptible piglet populations and supports its potential for direct clinical application as a feed additive in swine herds. However, a potential challenge for clinical application stems from the relatively low oral bioavailability and rapid intestinal metabolism of saponins, a key component class. To address this, future strategies could include the development of aerosolized formulations for direct respiratory targeting to enhance absorption, or the implementation of nanoparticle-based delivery systems to improve bioavailability, reduce dosage requirements, and mitigate potential hepatic and renal burden (Liu et al., 2025). Future investigations will employ molecular docking to identify MV-PRRSV protein interactions and conduct preclinical trials in porcine models to assess pharmacokinetics and efficacy. By integrating natural safety profiles with potent antiviral efficacy, MV represents a novel candidate molecule capable of redefining PRRSV prophylaxis and therapy.

## Conclusion

We have demonstrated that MV effectively inhibits PRRSV replication in both Marc-145 and PAM cells. To our knowledge, this study provides the first evidence that MV suppresses *in vitro* replication of swine-transmitted viral pathogens, with PRRSV serving as a representative model.

## Data Availability

The original contributions presented in the study are included in the article/supplementary material, further inquiries can be directed to the corresponding author.
